# Puerperal Peptonuria

**Published:** 1886-05

**Authors:** 


### Puerperal Peptonuria.

Dr. Ettore Truzzi has made 350 observations regarding this affection, and draws the following conclusions from his cases:

1. The appearance of peptones in the urine is of frequent occurrence during the first days after parturition, though it is not observed so often as Fischel would have us believe.

2. Puerperal peptonuria usually begins the second day after labor, increases on the third, reaches its maximum on the fourth, and ceases about the ninth day.

3. The pathology is obscure. Among the causes deserving attention are uterine involution and the constitution changes, which take place after parturition.—*Il Raccoglitore Med.*

